# COVID-19 laboratory preparedness and response in the Americas Region: Lessons learned

**DOI:** 10.1371/journal.pone.0253334

**Published:** 2021-06-29

**Authors:** Juliana Almeida Leite, Lionel Gresh, Andrea Vicari, Jean Marc Gabastou, Enrique Perez, Sylvain Aldighieri, SARINET laboratories Network, Jairo Mendez-Rico

**Affiliations:** Health Emergencies Department, Pan American Health Organization, Washington, District of Columbia, United States of America; University of Zambia, ZAMBIA

## Abstract

By the time the etiologic agent of the COVID-19 was identified as a novel coronavirus, no country in the Americas Region had laboratory capacity for detecting this new virus. A strategic multilevel approach with specific reagent purchase and delivery, regional trainings, in-country missions, and the provision of technical support was established for timely preparedness of national reference laboratories for SARS-CoV-2 detection. All countries should be prepared to timely detect any potential pandemic emerging agent. The rapid SARS-CoV-2 molecular detection implementation throughout the Americas showed the importance of an efficient and coordinated laboratory response for preparedness. Here we present how in 25 days the Americas Region went from no SARS-CoV-2 diagnostic capacity, to molecular detection fully implemented in 28 Member States, under the coordinated strategy of the Pan American Health Organization and collaborative work at regional and country level with national authorities and public health laboratories.

## Introduction

On 31 December 2019, China informed the World Health Organization (WHO) about a cluster of unusual severe pneumonia cases [1]. By early January, Chinese authorities officially announced a novel coronavirus as the etiologic agent responsible for this outbreak [2, 3]. First phylogenetic analysis indicated that this coronavirus was distinct from the severe acute respiratory syndrome (SARS-CoV) and Middle East respiratory syndrome (MERS-CoV) coronaviruses, being further classified within the same species as SARS-CoV and named SARS-CoV-2 by the International Committee on Taxonomy of Viruses (ICTV) [3, 4]. Simultaneously, WHO named the disease as COVID-19, for “coronavirus disease 2019” [5].

As the outbreak evolved with international spread, the disease clinical spectrum and its severity started to be elucidated. Additionally, the extent of human-to-human transmission in the community including healthcare facilities and the contribution of mild and asymptomatic were major concerns to be studied. Therefore, on 30 January 2020, the WHO declared COVID-19 outbreak a Public Health Emergency of International Concern (PHEIC) under International Health Regulations (IHR) [6]. The Emergency Committee recommendations stated all countries should be prepared for active surveillance and early detection of COVID-19 [6, 7].

In this context, the Pan American Health Organization (PAHO), WHO Regional Office for the Americas, worked to timely support Member States in preparing and responding to COVID-19. To coordinate its activities, PAHO activated the Incident Management System, which included a laboratory response team to support implementation of SARS-CoV-2 detection according to countries’ laboratory capacity. How the Americas Region went from no SARS-CoV-2 diagnostic capacity, to molecular detection fully implemented in 28 Member States in 25 days, under PAHO coordinated strategy and collaborative work at regional and country level with national authorities and public health laboratories is presented here.

## Materials and methods

### Existing laboratory capacity through WHO Global Influenza Surveillance and Response System

Countries in the Americas have been conducting influenza and other respiratory viruses’ surveillance as part of the WHO Global Influenza Surveillance and Response System (GISRS). Inside the influenza surveillance systems, the laboratory diagnosis is the only way to determine the specific aetiology of an Influenza-like-illness (ILI) or Severe Acute Respiratory Infection (SARI) case. Therefore, PAHO focused upon building capacity for virologic surveillance and improving the frequency, quantity and quality of the data that are shared through the GISRS network where the National Influenza Centres (NICs) and National Reference Laboratories serve as technical reference point surveillance within the country for influenza and other respiratory viruses.

Among the GISRS laboratory network in the Americas, 29 PAHO Member States and territories had molecular platforms, with real-time equipment for molecular detection of influenza and other respiratory viruses detection. Beside, PAHO has been working in strengthening existing or establishing new molecular platforms for emerging pathogens detection, through implementation of updated molecular diagnostic protocols, through provision of regional molecular diagnostic trainings, joint trainings with WHO Collaborating Centres, in-country missions for laboratory assessment, provision of reagents and supplies for molecular diagnostic sustainability. With all of this, the SARS-CoV-2 molecular implementation was mainly based on the NICs and national public health laboratories with a track record in the influenza laboratory surveillance.

### Laboratory preparedness for SARS-CoV-2 detection

On 20 January 2020, one day prior to the detection of the first case of COVID-19 in the Americas, laboratory technical guidance on SARS-CoV-2 detection was included in PAHO’s Epidemiological Update, published in English and Spanish [8]. This initial guidance was expanded into a specific document on “Laboratory Diagnosis of Novel Coronavirus (nCoV) infection” published first on 21 January, being routinely updated [9]. Additionally, “Interim laboratory biosafety guidelines for the handling and transport of samples associated with the novel coronavirus 2019” were released 28 January 2020. All these documents where published in English and Spanish and disseminated to Member States through IHR national focal points.

**Fig 1:**
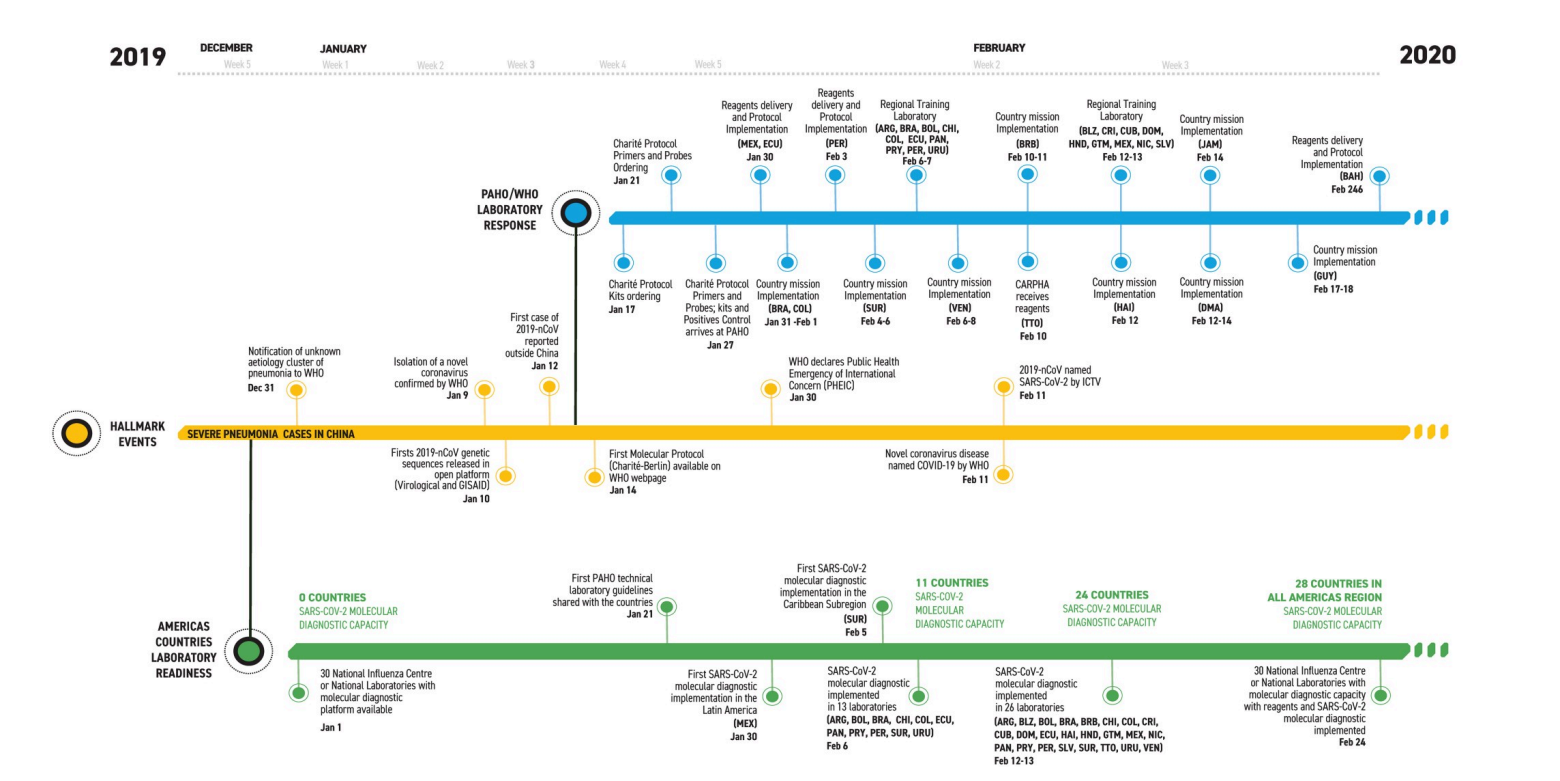
Timeline of the laboratory preparedness and response in the Americas Region to the COVID-19 outbreak, 31 December 2019 to 24 February 2020. Country codes: ARG-Argentina, BHS-Bahamas, BLZ-Belize, BOL-Bolivia, BRA-Brazil, BRB-Barbados, CHL-Chile, COL-Colombia, CRI-Costa Rica, CUB-Cuba, DMA-Dominica, DOM-Dominican Republic, ECU-Ecuador, GUY-Guyana, HAI-Haiti, HND-Honduras, GTM-Guatemala, JAM-Jamaica, MEX-Mexico, NIC-Nicaragua, PAN-Panama, PRY-Paraguay, PER-Peru, SLV-El Salvador, SUR-Suriname, TTO-Trinidad & Tobago, URU-Uruguay, VEN-Venezuela; CARPHA: Caribbean Public Health Agency. Data for Canada, French Guiana and United States not included in the timeline.

The strategy for implementation of SARS-CoV-2 detection involved a multilevel approach with specific reagent purchase and delivery, regional trainings, in-country missions, and the provision of continued technical support (Fig 1).

Laboratory response initiated with promptly synthesizing of primers and probes for SARS-CoV-2 screening targeting the envelope (E) gene and confirmation targeting the RNA-dependent RNA polymerase (RdRp) gene based on the protocol released and published by the Charité –Universitätsmedizin Berlin Institute of Virology, Germany [10]. Positive controls were obtained from MOLBIOL (USA) and kindly donated through the European Virus Archive—Global (EVAg). Also, commercially available kits with primers, probe and control based on the same protocol (LightMix® Modular SARS and Wuhan CoV E-gene and LightMix® Modular Wuhan CoV RdRP-gene, TIB MOLBIOL) were obtained. Reagents and critical material were procured by PAHO through regular acquisition mechanisms based on the RT-PCR reference protocol and from reliable providers. To ensure a harmonized process, most of the material was delivered at PAHO Head Quarters for a strategic stockpile that was distributed directly to laboratories or thorough PAHO Country Offices using certified couriers with cold-chain granted when needed. Additional reagents were provided to training host laboratory (InDRE in Mexico, and Fiocruz in Brazil) and used during regional trainings.

SARS-CoV-2 molecular diagnostic implementation in the Americas Region started 30 January 2020 with in-country missions of PAHO virology experts to Brazil and Suriname, being followed by sub regional trainings and additional missions for providing capacitation and assistance to participants and laboratory staff on SARS-CoV-2 molecular diagnostic implementation and reagent delivery. Additional technical support was provided to the countries either by discussion of questions and challenges raised during the regional trainings as well as though individual meetings and troubleshooting sections requested either by the Ministries of Health or the National Laboratories.

## Results and discussion

The first sub-regional laboratory training was held in Brazil on 6–7 February 2020 for eight South American countries (Argentina, Bolivia, Chile, Colombia, Ecuador, Paraguay, Peru and Uruguay) and Panama. The second in Mexico on 12–13 February for six Central American countries (Belize, Costa Rica, El Salvador, Honduras, Guatemala, and Nicaragua), Cuba, Dominican Republic, and Mexico. All participating countries were trained on Charité screening and confirmatory assays and received reagents for implementation in their respective countries, that included primers, probes and positive controls.

Parallel in-country missions for SARS-CoV-2 molecular diagnostic implementation as well as reagent delivery were conducted in Venezuela, Haiti, Barbados, Jamaica, Dominica, and Guyana (Fig 1). Likewise, on 05 February 2020 reagents for Charité protocol were shipped to the Medical Microbiology Laboratory at Caribbean Public Health Agency (CARPHA), which serves as NIC for the Caribbean Subregion. In-country missions focused diagnostic implementation at the national laboratories with on-site PAHO training and guidance, while individual countries implementation was held by skilled personal from the laboratory through remote PAHO assistance.

Concurrently, PAHO provided molecular diagnostic reagents and critical material, as laboratory supplies, enzymes and extraction kits, to ensure the continuity of the COVID-19 laboratory surveillance. In all laboratories, implementation was successful and expected results were obtained for positive and negative controls, generating reliable results.

As anticipated, some challenges and limitations were encountered during the course of implementation of the SARS-CoV-2 molecular detection in the region. Some countries do not have a fast procurement system and availability of certain reagents including oligonucleotide synthesis in-country, so PAHO worked with the stock-pile strategy for providing auxiliary reagents and supplies. Likewise, flight restrictions impacted the in-country missions, so meetings and support activities successfully migrated to virtual platform. As always, customs clearance process has generated delays on timely material delivery to the National Laboratories. PAHO worked supporting the countries Ministry of Health for expediting clearance. Optimizing customs process which involve reagents and supplies for responding to a health emergency, with no affecting of fiscal functions or customs tariff, would enable a more effective and timely response to the health emergency. Prioritization of customs clearance for these materials should be considered in the countries’ health contingency plans.

By the time of the first notification of the unknown aetiology cluster of pneumonia in Wuhan to WHO and the release of the first SARS-CoV-2 molecular detection protocol, the Americas Region had no SARS-CoV-2 diagnostic capacity. By February 12, less than 15 days after declaration of COVID-19 PHEIC, 75% (26/30) of the laboratories in the Region had successfully implemented the SARS-CoV-2 detection.

By February 24, 28 of the 29 countries had the SARS-CoV-2 molecular diagnostic timely implemented with support of the Laboratory Response Team of the PAHO Health Emergency Department under coordination of the COVID-19 Incident Manager. In terms of regional capacity, it is important to mention that the United States of America and Canada timely developed and implemented their own in-house protocols for SARS-CoV-2 molecular detection. French Guiana timely started diagnostic based on Charité and Hong Kong protocols through the Institute Pasteur International Network. Thus, the full implementation of SARS-CoV-2 molecular detection in all the laboratory network in the Americas was achieved 25 days after the COVID-19 PHEIC declaration and 16 days before the WHO declaration of COVID-19 Pandemic on March 11 (Table 1).

**Table 1:** COVID-19 national laboratory readiness in the Americas Region, February 2020.

Country	COVID-19 Diagnostic Laboratory	National Influenza Center	Currently testing for COVID-19	Staff trained for COVID-19 PCR	Has COVID-19 Reagents	COVID-19 protocol in use	COVID-19 reagent in use^a^^,^^b^	Has access to ancillary reagents^c^
Argentina	Instituto Nacional de Enfermedades Infecciosas -ANLIS C.G.Malbran	Yes	Yes	Yes	Yes	Charité - Berlin Protocol	Primers, Probes and positive controls for E gene and RdRP gene and TIB BioMol kits	Yes
Brazil	Fundação Oswaldo Cruz (FIOCRUZ)	Yes	Yes	Yes	Yes	Charité - Berlin Protocol	Primers, Probes and positive controls for E gene and RdRP gene and Kits provided by PAHO and by BRA MoH	Yes
	Instituto Adolfo Lutz (IAL)	Yes	Yes	Yes	Yes	Charité - Berlin Protocol	Primers, Probes and positive controls for E gene and RdRP gene and Kits provided by PAHO and by BRA MoH	Yes
	Instituto Evandro Chagas (IEC)	Yes	Yes	Yes	Yes	Charité - Berlin Protocol	Primers, Probes and positive controls for E gene and RdRP gene and Kits provided by PAHO and by BRA MoH	Yes
Bahamas	Ministry of Health Reference Laboratory	No	Yes	Yes	Yes	Charité - Berlin Protocol	TIB Bio Mol Kit for E gene and RdRP gene provided by PAHO	Yes
Barbados	Best-Dos Santos Public Health Laboratory	No	Yes	Yes	Yes	Charité - Berlin Protocol	TIB Bio Mol Kit for E gene and RdRP gene provided by PAHO	Yes
Belize	Central Medical Laboratory	No	Yes	Yes	Yes	Charité - Berlin Protocol	TIB Bio Mol Kit for E gene and RdRP gene provided by PAHO	Yes
Bolivia	Centro Nacional de Enfermedades Tropicales (CENETROP)	Yes	Yes	Yes	Yes	Charité - Berlin Protocol	TIB Bio Mol Kit for E gene and RdRP gene provided by PAHO	Yes
Canada	National Microbiology Laboratory Health Canada Canadian Science Center for Human and Animal Health	Yes	Yes	Yes	Yes	*in house*	-	Yes
Chile^d^	Instituto de Salud Pública de Chile (ISPC)	Yes	Yes	Yes	Yes	Charité - Berlin Protocol	TIB Bio Mol Kits provided by PAHO; Primers, and Probes from ISPC and positive controls provided bay PAHO	Yes
Colombia	Instituto Nacional de Salud (INS)	Yes	Yes	Yes	Yes	Charité - Berlin Protocol	Primers, Probes and positive controls for E gene and RdRP gene provided by PAHO	Yes
Costa Rica	Instituto Costarricense de Investigación y Enseñanza en Nutrición y Salud (INCIENSA)	Yes	Yes	Yes	Yes	Charité - Berlin Protocol	TIB Bio Mol Kit for E gene and RdRP gene provided by PAHO	Yes
Cuba	Instituto de Medicina Tropical "Pedro Kourí" (IPK)	Yes	Yes	Yes	Yes	Charité - Berlin Protocol	TIB Bio Mol Kit for E gene and RdRP gene provided by PAHO	Yes
Dominica	Princess Margaret Hospital Medical Laboratory	No	Yes	Yes	Yes	Charité - Berlin Protocol	TIB Bio Mol Kit for E gene and RdRP gene provided by PAHO	Yes
Dominican Republic	Laboratorio Nacional de Salud Pública Dr. Defillo (LNSPDD)	Yes	Yes	Yes	Yes	Charité - Berlin Protocol	TIB Bio Mol Kit for E gene and RdRP gene provided by PAHO	Yes
Ecuador	Instituto Nacional de Investigación en Salud Pública (INISP)	Yes	Yes	Yes	Yes	Charité - Berlin Protocol	TIB Bio Mol Kit for E gene and RdRP gene provided by PAHO	Yes
El Salvador	Laboratorio Central Ministerio de Salud Publica "Dr Max Bloch"	Yes	Yes	Yes	Yes	Charité - Berlin Protocol	TIB Bio Mol Kit for E gene and RdRP gene	Yes
French Guiana	Institut Pasteur de la Guyane	Yes	Yes	Yes	Yes	Hong Kong University and Charité –Berlin protocols	-	Yes
Guatemala	Laboratorio Nacional de Salud (LNS)	Yes	Yes	Yes	Yes	Charité - Berlin Protocol	TIB Bio Mol Kit for E gene and RdRP gene provided by PAHO	Yes
Guyana	National Public Health Reference Laboratory	No	Yes	Yes	Yes	Charité - Berlin Protocol	TIB Bio Mol Kit for E gene and RdRP gene provided by PAHO	Yes
Haiti	Laboratoire National de Santé Publique (LNSP)	Yes	Yes	Yes	Yes	Charité - Berlin Protocol	TIB Bio Mol Kit for E gene and RdRP gene provided by PAHO	Yes
Honduras	Laboratorio Nacional de Vigilancia de la Salud (LNV)	Yes	Yes	Yes	Yes	Charité - Berlin Protocol	TIB Bio Mol Kit for E gene and RdRP gene provided by PAHO	Yes
Jamaica	Virology Laboratory University of the West Indies	Yes	Yes	Yes	Yes	Charité - Berlin Protocol	TIB Bio Mol Kit for E gene and RdRP gene provided by PAHO	Yes
Mexico	Instituto de Diagnóstico y Referencia Epidemiologicos (InDRE)	Yes	Yes	Yes	Yes	Charité - Berlin Protocol	Primers, Probes locally synthesized for E gene and RdRP gene and positive controls provided by PAHO	Yes
Nicaragua	Centro Nacional de Diagnóstico y Referencia (CNDR)	Yes	Yes	Yes	Yes	Charité - Berlin Protocol	TIB Bio Mol Kit for E gene and RdRP gene provided by PAHO	Yes
Panama	Instituto Conmemorativo Gorgas de Estudios de la Salud (ICGES)	Yes	Yes	Yes	Yes	Charité - Berlin Protocol	Primers, Probes locally synthesized for E gene and RdRP gene and positive controls provided by PAHO	Yes
Paraguay	Laboratorio Central de Salud Publica (LCSP)	Yes	Yes	Yes	Yes	Charité - Berlin Protocol	Primers, Probes locally synthesized for E gene and RdRP gene and positive controls provided by PAHO	Yes
Peru	Instituto Nacional de Salud (INS)	Yes	Yes	Yes	Yes	Charité - Berlin Protocol	TIB Bio Mol Kit for E gene and RdRP gene	Yes
Suriname	Laboratorium Bureau Openbare Gezondheidszorg (BOG Central Laboratory)	No	Yes	Yes	Yes	Charité - Berlin Protocol	Primers, Probes and positive controls for E gene and RdRP gene provided by PAHO	Yes
Trinidad and Tobago	Caribbean Public Health Agency (CARPHA)	Yes	Yes	Yes	Yes	Charité - Berlin Protocol	Primers, Probes and positive controls for E gene and RdRP gene provided by PAHO	Yes
Uruguay	Departamento de Laboratorio de Salud Publica (DLSP)	Yes	Yes	Yes	Yes	Charité - Berlin Protocol	Primers, Probes locally synthesized for E gene and RdRP gene and positive controls provided by PAHO	Yes
United States of America	Respiratory Viruses Diagnostic Laboratory, US-CDC	No	Yes	Yes	Yes	*CDC Protocol*	-	Yes
Venezuela	Instituto Nacional de Higiene Rafael Rangel (INHRR)	Yes	Yes	Yes	Yes	Charité - Berlin Protocol	TIB Bio Mol Kit for E gene and RdRP gene provided by PAHO	Yes

^a^Primers, probes and positive controls for E gene and RdRP gene for up to 2,000 tests provided by PAHO.

^b^TIB BIOMOL kits for up to 96 tests provided by PAHO.

^c^Ancillary reagents for molecular diagnostic refers to reagents as enzymes and extraction kits for the sustainability of the implemented protocol in the countries, since these additional reagents demand was not predicted.

^d^At the moment of the submission, Chile was using other commercial protocol.

As all countries should be prepared to detect the SARS-CoV-2, the rapid implementation of SARS-CoV-2 molecular detection showed the importance of an efficient and coordinated laboratory response for preparing the Americas Region for a pandemic (Table 2). The implementation of the SARS-CoV-2 detection throughout the Americas was possible due to four main points: (1) collaborative work at regional and country level, among PAHO/WHO, national authorities and national public health laboratories; (2) fast planning of the strategic response; (3) efficient logistic for procurement and distribution of necessary reagents, including strategic stockpile at PAHO headquarters; and (4) field missions for in-country and sub regional trainings. Importantly, regional efforts to strengthen the surveillance of influenza, other respiratory viruses, and emerging and re-emerging viruses in the past decade have laid the ground for timely and widespread implementation. The main limitation of the response at that moment was the lack of proficiency panels to independently verify performance of trained laboratories. However, proficiency panels under WHO coordination were available in April 2020 and distributed in the Region to assess laboratory performance, with 94% (28/30) of the participating laboratories having 100% score.

**Table 2:** Summary of main lessons learnt on laboratory response for preparing to a pandemic.

Action	Lesson learnt
Planning	Fast planning of a strategic response is essential for an efficient and timely laboratory readiness to respond to a public health event of international concern before it becomes a pandemic.
Collaboration	A collaborative work at regional and country level, among public health agencies, national authorities and national public health laboratories is critical for the laboratory’s preparedness and appropriate response during the pandemic phase.
Logistic	Efficient logistic for obtaining and distribution of necessary reagents and critical material, including strategic stockpile made possible the readiness and timely implementation of molecular detection protocols in countries as well as the continuous support for maintenance of the testing sustainability in countries.
Missions and trainings	Field missions in-country and sub regional trainings were fundamental for expediting the laboratory preparedness and response.

Under the PAHO Incident Management System, there are currently three critical actions for the COVID-19 response in the Americas: (1) save lives by reorganizing health services, maintaining infection prevention and control and optimizing clinical management; (2) protect health care workers at work and in the community and (3) slow spread of COVID-19 by detecting and isolating cases, quarantining contacts and implementing social distancing and travel-related measures [11]. Epidemiologic and laboratorial surveillance is key for these actions. Therefore, laboratory readiness is an important pillar of this response. The COVID-19 pandemic impacted most of the countries world-wide, including the laboratories testing capacity with needs of reagents, trainings, human resource and all elements that are involved on laboratory readiness for a response. Thus, it is important to continue working together with the designated national laboratories for strengthening and maintenance of the laboratory’s response capacity and sustainability of the SARS-CoV-2 detection.

## Conclusion

Laboratory readiness is a key tenet for rapid response to an emerging disease with potential to become a pandemic. Fast planning of a strategic response was essential for a timely laboratory preparedness. Collaboration at regional and country level, among public health agencies, national authorities and national public health laboratories is critical for the laboratory’s preparedness and appropriate response during the pandemic phase. Efficient logistic for obtaining and distribution of necessary reagents and critical material, including strategic stockpile made possible the readiness and timely implementation of SARS-CoV-2 molecular detection protocols in the countries of the Americas Region as well as the continuous support for maintenance of the testing sustainability in countries. Field missions in-country and sub regional trainings were also fundamental for expediting the laboratory preparedness and response. PAHO continues to collaborate at regional and country level with national authorities and public health laboratories for leveraging laboratory readiness in the Americas Region for responding to the COVID-19 pandemic and any emerging viral diseases.
